# Association of Longitudinal Values of Glycated Hemoglobin With Cardiovascular Events in Patients With Type 2 Diabetes and Multivessel Coronary Artery Disease

**DOI:** 10.1001/jamanetworkopen.2019.19666

**Published:** 2020-01-22

**Authors:** Paulo Cury Rezende, Mark Andrew Hlatky, Whady Hueb, Rosa Maria Rahmi Garcia, Luciano da Silva Selistre, Eduardo Gomes Lima, Cibele Larrosa Garzillo, Thiago Luis Scudeler, Gustavo Andre Boeing Boros, Fernando Faglioni Ribas, Carlos Vicente Serrano, Jose Antonio Franchini Ramires, Roberto Kalil Filho

**Affiliations:** 1 Instituto do Coração (InCor), Hospital das Clinicas, Faculdade de Medicina, Universidade de São Paulo, São Paulo, Brazil; 2 Stanford University School of Medicine, Stanford, California; 3 Universidade Caxias do Sul, Caxias do Sul, Brazil

## Abstract

**Importance:**

Glycated hemoglobin (HbA_1c_) values are used to guide glycemic control, but in patients with type 2 diabetes and multivessel coronary artery disease (CAD), the association of the longitudinal values of HbA_1c_ with cardiovascular outcomes is unclear.

**Objective:**

To assess whether longitudinal variation of HbA_1c_ is associated with cardiovascular events in long-term follow-up among patients with diabetes and multivessel CAD.

**Design, Setting, and Participants:**

This cohort study included 888 patients with type 2 diabetes and multivessel CAD in the Medicine, Angioplasty, or Surgery Study (MASS) Registry of the Heart Institute of the University of São Paulo from January 2003 to December 2007. Data were analyzed from January 15, 2018, to October 15, 2019.

**Exposure:**

Longitudinal HbA_1c_ values.

**Main Outcomes and Measures:**

The combined outcome of all-cause mortality, myocardial infarction, and ischemic stroke.

**Results:**

Of 888 patients with type 2 diabetes and multivessel CAD, 725 (81.6%; median [range] age, 62.4 [55.7-68.0] years; 467 [64.4%] men) had complete clinical and HbA_1c_ information during a median (interquartile range) follow-up period of 10.0 (8.0-12.3) years, with a mean (SD) of 9.5 (3.8) HbA_1c_ values for each patient. The composite end point of death, myocardial infarction, or ischemic stroke occurred in 262 patients (36.1%). A 1-point increase in the longitudinal value of HbA_1c_ was significantly associated with a 14% higher risk of the combined end point of all-cause mortality, myocardial infarction, and ischemic stroke (hazard ratio, 1.14; 95% CI, 1.04-1.24; *P* = .002) in the unadjusted analysis. After adjusting for baseline factors (ie, age, sex, 2-vessel or 3-vessel CAD, initial CAD treatments, ejection fraction, and creatinine and low-density lipoprotein cholesterol levels), a 1-point increase in the longitudinal value of HbA_1c_ was associated with a 22% higher risk of the combined end point (hazard ratio, 1.22; 95% CI, 1.12-1.35; *P* < .001).

**Conclusions and Relevance:**

Longitudinal increase of HbA_1c_ was independently associated with higher rates of cardiovascular events in patients with type 2 diabetes and multivessel CAD.

## Key Points

**Question:**

Are longitudinal glycated hemoglobin values associated with cardiovascular events in patients with type 2 diabetes and stable multivessel coronary artery disease?

**Findings:**

In this cohort study of 725 patients with type 2 diabetes and multivessel coronary artery disease, a 1-point increase in glycated hemoglobin values during follow-up was independently associated with higher risk of the combined outcome of death, myocardial infarction, or ischemic stroke, after adjustment for baseline clinical factors.

**Meaning:**

Longitudinal increase of glycated hemoglobin was associated with higher rates of cardiovascular events in patients with type 2 diabetes and multivessel coronary artery disease, and the mechanisms underlying this association require further investigation.

## Teaser

This cohort study examines whether longitudinal variation of glycated hemoglobin (HbA_1c_) is associated with cardiovascular events in patients with diabetes and multivessel coronary artery disease (CAD).

## Introduction

Glucose control among patients with diabetes is primarily guided by the periodic assessment of glycated hemoglobin (HbA_1c_) levels. Strict control of diabetes, indicated by lower HbA_1c_ levels, has been associated with reduced microvascular complications, including nephropathy, retinopathy, and peripheral neuropathy.^1,2,3^ Strict control of diabetes is less associated with macrovascular events, such as myocardial infarction (MI) or ischemic stroke. Although some studies^4^ have shown that lower HbA_1c_ levels were associated with lower rates of cardiovascular mortality and MI, landmark randomized clinical trials^5,6^ have not found significant decreases in cardiovascular end points with intensive glucose control. Additionally, the Action to Control Cardiovascular Risk in Diabetes (ACCORD) trial^7^ was prematurely halted because of higher rates of cardiac mortality in the group of patients with target HbA_1c_ levels lower than 6.0% (to convert to proportion of total hemoglobin, multiply by 0.01).^7^

The increased cardiovascular mortality reported by the ACCORD trial remains unexplained; exploratory analyses^8^ suggested it may have been because of the use of multiple combinations of glucose-lowering medications in the intensive arms, weight gain, or drug-drug interactions. It is also possible that increased rates of severe hypoglycemia in the intensive therapy group may have contributed to the increased cardiovascular mortality rate because hypoglycemia has been associated with elevated troponin levels, a marker of myocardial damage.^9^ However, chronic hyperglycemia is associated with a chronic state of systemic inflammation, modulated by reactive oxygen species and advanced glycation end products, which may lead to vascular damage.^10^

Because hyperglycemia and hypoglycemia may both have adverse cardiovascular consequences, variability in glucose control may have unfavorable effects, even when an individual’s average HbA_1c_ levels are acceptable. Excess variability in glucose and HbA_1c_ levels that results from chronic fluctuations over time may indicate increased cardiovascular risk in the diabetic population,^11,12^ although the possibility has been controversial.^12,13,14,15^

Patients with established coronary artery disease (CAD) may be particularly susceptible to poor glucose control. The few studies of longitudinal HbA_1c_ variation in patients with diabetes and CAD have assessed a limited number of HbA_1c_ measures, had short follow-up, or were based on heterogeneous populations. The present study aimed to assess whether diabetes control, based on HbA_1c_ follow-up values, is associated with the occurrence of macrovascular events during a long-term follow-up period in a carefully studied cohort of patients with type 2 diabetes with multivessel CAD.

## Methods

### Study Design and Population

The study population consisted of patients with type 2 diabetes enrolled in the Medicine, Angioplasty, or Surgery Study (MASS) Registry of the Heart Institute of the University of São Paulo from January 2003 to December 2007. Because of the retrospective nature of this study, a waiver of ethical review and informed consent was granted by the ethics committee of the Heart Institute (InCor), University of São Paulo Medical School. This study followed the Strengthening the Reporting of Observational Studies in Epidemiology (STROBE) reporting guideline.

Patients were eligible for the MASS Registry if they had multivessel CAD, stable angina symptoms, or documented myocardial ischemia and were candidates for 1 of 3 treatment modalities for multivessel CAD, as follows: medical therapy, percutaneous coronary intervention (PCI), or coronary artery bypass grafting (CABG). All clinical and laboratory information has been tracked prospectively since trial registration and recorded in study-specific databases.

Multivessel CAD was confirmed by coronary angiography demonstrating obstructive lesions in at least 2 branch vessels with at least 70% obstruction. Type 2 diabetes was confirmed by the following criteria: use of insulin and/or oral antihyperglycemic agents, 2 fasting glucose levels of at least 126 mg/dL (to convert to millimoles per liter, multiply by 0.0555), an HbA_1c_ level of at least 6.5%, a random glucose level of at least 200 mg/dL, or a 2-hour plasma glucose of at least 200 mg/dL during an oral glucose tolerance test. Left ventricular function was assessed by echocardiography and was considered preserved if the ejection fraction was at least 0.35. Patients were excluded from the MASS Registry if they had experienced an acute coronary syndrome in the last 3 months, had a serum creatinine level greater than 2.0 mg/dL (to convert to micromoles per liter, multiply by 88.4), hepatic dysfunction, active cancer, or life expectancy of less than 2 years. Additional exclusions for the present study were an ejection fraction of 35% or less at baseline, incomplete clinical data regarding cardiovascular outcomes, or lack of HbA_1c_ measurements during follow-up.

All patients were observed at the Heart Institute of the Clinical Hospital of the University of São Paulo by outpatient visits every 6 months and were placed in optimal medical therapy to reach specific treatment goals. Rigorous monitoring of glycemic control aimed to achieve an HbA_1c_ level less than 7.0% without hypoglycemic events. The goals of medical therapy also included systolic arterial pressure of 140 mm Hg or less, diastolic arterial pressure of 90 mmHg or less, and low-density lipoprotein cholesterol (LDL-C) levels of 70 mg/dL or less (to convert to millimoles per liter, multiply by 0.0259).

### Measurement of HbA_1c_

We measured HbA_1c_ by the immunoturbidimetric method, certified by the National Glycohemoglobin Standardization Program, with a normal range of 4.5% to 6.2%. All HbA_1c_ measurements were recorded in a specific database and used in this analysis. All recorded HbA_1c_ measures during follow-up were assessed until the occurrence of the first composite clinical end point.

### Clinical Events

Patients were prospectively assessed for cardiovascular events after entry into the MASS Registry. All events were classified by review of death certificates, family information, and hospital records. The primary outcome of this analysis was the composite end point of death from any cause, MI, or ischemic stroke. The follow-up for the primary outcome was censored on December 31, 2016. Myocardial infarction was defined as an acute episode of chest pain, associated with electrocardiographic evidence of myocardial ischemia and elevated cardiac biomarker, creatine kinase–MB, or troponin levels above cutoff values. Stroke was defined as an acute episode of focal neurologic dysfunction, confirmed by brain imaging (ie, computed tomography or magnetic resonance imaging). Patients underwent new revascularizations if they developed limiting angina or an acute coronary syndrome, with coronary anatomical features feasible for angioplasty or surgery.

### Statistical Analysis

Data were analyzed from January 15, 2018, to October 15, 2019. Categorical variables are reported as absolute numbers and percentages, and continuous variables are reported as means and SDs or as medians and ranges. Categorical data were compared using the χ^2^ test or the Fisher exact test. Continuous data were compared using the Wilcoxon rank sum test. Time-to-event outcomes are presented using Kaplan-Meier survival curves and number at risk tables.

Measurements of HbA_1c_ were truncated before the occurrence of the clinical end point. A joint model^16,17,18^ was used to correlate longitudinal data (ie, HbA_1c_ measurements) with the occurrence of clinical events. In this model, both linear mixed-effects and Cox models were used, and a spline–proportional hazard–Gauss-Hermite method was assumed to specify the type of baseline risk function of the survival submodel, which was assumed as spline-approximation of the log baseline risk function. Patients were excluded from these analyzes if they only had HbA_1c_ measures after the clinical end point or if they had missing data on clinical covariates. The linear mixed-effects regression was used to model the evolution of HbA_1c_ levels until the clinical event. Both the intercepts and the slopes were used as random-effects terms, assuming that the rate of change in HbA_1c_ would be different between patients. To allow for a flexible specification of the patient-specific longitudinal trajectories, a natural cubic spline effect for time was assumed with 2 internal knots placed at the 33.3% and 66.7% percentiles of the follow-up times in the linear mixed-effects model. Additionally, a separate indicator variable of the baseline measurement was included to capture sudden changes in HbA_1c_ values in the first year of follow-up. The lme function of package nlme in R software (R Project for Statistical Computing) was used to fit the model and the restricted maximum likelihood method was used to estimate the model parameters. A Cox regression was used to model the timing to the occurrence of the predefined composite primary event (ie, the first occurrence of all-cause death, MI, or ischemic stroke), first unadjusted and then adjusted for baseline clinical covariates (ie, age, sex, ejection fraction, number of coronary artery vessels diseased, initial CAD treatment, and creatinine and LDL-C levels).

Sensitivity analyses were also performed, analyzing distinct models. A second model (model B) was fit using time as a linear term, and a third model was also constructed adding a quadratic term of time to the first model (model C). Analysis of variance tests were used to compare the results of the joint models.

All tests were 2-tailed, and *P* < .05 was considered statistically significant. All analyses were performed using R software version 3.5.3 (R Project for Statistical Computing).

## Results

The MASS Registry enrolled 888 patients with type 2 diabetes and multivessel CAD. Of these, 140 patients (15.8%) had no HbA_1c_ information during follow-up, 7 patients (0.8%) had incomplete clinical follow-up data, and 16 patients (1.8%) had an ejection fraction of 0.35 or less. The final study population comprised 725 patients, who were observed for a median (IQR) of 10.0 (8.0-12.3) years. A total of 6876 HbA_1c_ measurements were used in this analysis, with a mean (SD) of 9.5 (3.8) HbA_1c_ values for each patient.

### Baseline Characteristics

The baseline characteristics of the 725 patients are shown in Table 1. The median (range) age was 62.4 (55.7-68.0) years, and 467 patients (64.4%) were men. Approximately 70% of the population consisted of patients with 3-vessel CAD (442 [68.4%]), ejection fraction was preserved (median, 65%; range 60%-70%), and median (range) HbA_1c_ levels at baseline were 7.5% (6.4%-9.2%). The baseline characteristics of the 725 patients included in this analysis were compared with the 163 patients excluded from this analysis (eTable 1 in the Supplement). The main clinical characteristics, such as age, ejection fraction, number of CAD vessels with obstructive lesions, and initial CAD therapies, were similar between the 2 groups.

**Table 1.** Baseline Characteristics of Study Population

Characteristic	No. (%) (N = 725)
Age, median (range), y	62.4 (55.7-68.0)
Men	467 (64.4)
Hypertension	534 (75.5)
Smoking status	
Current	119 (16.9)
Never	351 (50.0)
Former	232 (33.1)
CKD, ie, creatinine level >1.5 mg/dL	37 (5.1)
Ejection fraction, median (range), %	65.0 (60.0-70.0)
CAD	
2-Vessel	204 (31.6)
3-Vessel	442 (68.4)
Initial CAD treatment	
Medical therapy	203 (28.1)
CABG	328 (45.4)
PCI	192 (26.5)
LDL-C level, median (range), mg/dL	113 (89-144)
Baseline HbA_1c_ level, median (range), %	7.50 (6.40-9.20)
Creatinine level, median (range), mg/dL	1.00 (0.89-1.20)

Abbreviations: CABG, coronary artery bypass grafting; CAD, coronary artery disease; CKD, chronic kidney disease; HbA_1c_, glycated hemoglobin; LDL-C, low-density lipoprotein cholesterol; PCI, percutaneous coronary intervention.

SI conversion factors: To convert LDL-C to mmol/L, multiply by 0.0259; HbA_1c_ to proportion of total hemoglobin, multiply by 0.01; and creatinine to μmol/L, multiply by 88.4.

### Cardiovascular Events

Of 725 patients, 204 (28.1%) died during follow-up: 95 (46.6%) due to cardiovascular causes, 75 (36.8%) due to noncardiovascular causes, and 34 (16.6%) due to unknown causes. The annual all-cause mortality rate was 2.8%, and the annual cardiovascular mortality rate was 1.3%. Nonfatal MI occurred in 82 patients (11.3%), and stroke occurred in 41 patients (5.6%). During follow-up, 57 patients (7.9%) underwent CABG and 76 (10.5%) underwent PCI. The composite end point of death, MI, or ischemic stroke occurred in 262 patients (36.1%). The Figure shows a Kaplan-Meier survival curve of all 725 patients with diabetes during 10-year follow-up for the primary end point.

**Figure.**
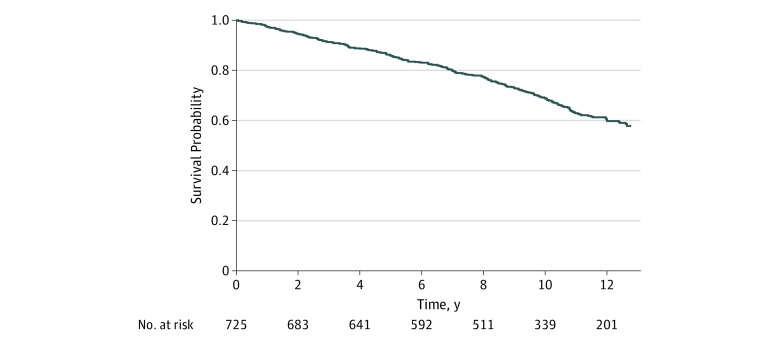
Kaplan-Meier Survival Curve for the Combined Event Rates of Death, Myocardial Infarction, and Ischemic Stroke in Patients With Diabetes and Multivessel Coronary Artery Disease

### Cardiovascular Events and HbA_1c_ Longitudinal Variation

The results of the unadjusted joint model show that a 1-point increase in HbA_1c_ level during follow-up was associated with a hazard ratio (HR) of 1.14 (95% CI, 1.04-1.24; *P* = .002) in the risk of the primary combined end point. After adjusting for baseline covariates, a 1-point increase in HbA_1c_ was associated with an HR of 1.22 (95% CI, 1.12-1.35; *P* < .001) (Table 2).

**Table 2.** Results of the Joint Models of the Association of HbA_1c_ Variation Over Time With the Occurrence of the Composite End Point^a^

Variable	Model			
	Unadjusted		Adjusted^b^	
	HR (95% CI)^c^	*P* Value	HR (95% CI)^c^	*P* Value
HbA_1c_ level, %	1.14 (1.04-1.24)	.002	1.22 (1.12-1.35)	<.001
Age, y	NA	NA	1.04 (1.02-1.07)	<.001
Male sex	NA	NA	0.84 (0.65-1.14)	.20
Ejection fraction, %	NA	NA	0.97 (0.96-0.99)	.001
3-Vessel CAD vs 2-vessel CAD	NA	NA	1.07 (0.83-1.32)	.58
Initial CAD therapies	NA	NA	0.73 (0.63-0.89)	<.001
Creatinine level, mg/dL	NA	NA	1.63 (0.95-2.73)	.07
LDL-C level, mg/dL	NA	NA	1.00 (0.98-1.02)	.24

Abbreviations: CAD, coronary artery disease; HbA_1c_, glycated hemoglobin; HR, hazard ratio; LDL-C, low-density lipoprotein cholesterol; NA, not applicable.

^a^ The composite end point was the first occurrence of death, myocardial infarction, or ischemic stroke.

^b^ Model adjusted for age, sex, ejection fraction, 2-vessel or 3-vessel CAD, initial CAD therapy, and creatinine and LDL-C levels.

^c^ For continuous variables, HRs are for a 1-unit increase in the variable.

Table 2 also shows the HRs of all covariates included in the adjusted model. Besides HbA_1c_ longitudinal levels, age (HR, 1.04; 95% CI, 1.02-1.07; *P* < .001), ejection fraction (HR, 0.97; 95% CI, 0.96-0.99; *P* = .001), and initial CAD therapies (HR, 0.73; 95% CI, 0.63-0.89; *P* < .001) showed independent associations with the composite primary end point.

All the models (ie, unadjusted, adjusted, model B, and model C) showed a statistically significant association of a 1-point variation in HbA_1c_ values with the higher risk of the combined events. These results are shown in eTable 2 in the Supplement, and the comparison of the results of these joint models is shown in eTable 3 in the Supplement.

## Discussion

This study showed that the increase in HbA_1c_ values at a particular time during follow-up was independently associated with higher rates of cardiovascular events among patients with type 2 diabetes and multivessel CAD. The results showed that a 1-point increase in HbA_1c_ values was independently associated with a 22% increase in the risk of the combined end point of death, MI, or ischemic stroke. Thus, this study suggested that higher HbA_1c_ values were associated with clinical events even after adjusting for important baseline clinical factors, such as age, ejection fraction, and initial CAD therapies. The statistical adjustments for these covariates and the use of a joint model reinforce the association of HbA_1c_ values with outcomes.

These results support the idea that glycemic control may have a prognostic association with cardiovascular events. Thus, HbA_1c_ control may not only influence the development of microvascular complications but also be associated with macrovascular events.

Although all patients underwent rigorous control of HbA_1c_ during follow-up, performed by the same group of physicians using similar treatment strategies, it is possible that the patients with higher fluctuations of glycemia and, consequently, of HbA_1c_ had more severe diabetes, less pancreatic reserve, and, thus, more difficulty controlling glycemia. Moreover, it is also possible this group of patients had lower adherence to the treatment.

Some mechanisms may be involved in the association of HbA_1c_ variation with cardiovascular events. Glycemic variation has been associated with superoxide overproduction^19,20^ and with an increase in inflammatory cytokines and macrophage adhesion to endothelial cells.^21^ Moreover, patients with higher variation in glucose parameters are probably more likely to have more frequent episodes of both hyperglycemia and hypoglycemia. The systemic consequences of hyperglycemia have been postulated and include stimulation of inflammatory cascades as well as microvascular and macrovascular damage.^10^

Additionally, among other alterations associated with hypoglycemia, these episodes have been associated with electrocardiographic signs of myocardial ischemia,^22^ disarrangements in ventricular repolarization,^23^ and arrhythmias^24^ and may possibly trigger myocardial injury and the release of cardiac biomarkers, such as troponin.^9^ These pathological disorders might explain the association of HbA_1c_ variation with higher rates of cardiac events.

The findings of other trials^12,13,14^ of patients with type 2 diabetes who had risk factors, macrovascular disease, or microvascular disease have indicated similar results. A substudy of the Action in Diabetes and Vascular Disease: Preterax and Diamicron Modified Release Controlled Evaluation (ADVANCE) trial^12^ showed HbA_1c_ variability to be an independent predictor of all-cause mortality or future macrovascular events in patients with diabetes and risk factors for or evidence of vascular disease. A 2013 Italian study^13^ also showed that HbA_1c_ variability was more strongly associated with all-cause mortality than mean HbA_1c_ values in a general population of patients with type 2 diabetes. On the other hand, a 2018 analysis^14^ of patients in the Veterans Affairs Diabetes Trial found that glucose variability but not HbA_1c_ variability was associated with cardiovascular disease. The authors found an independent association of fasting glycemia variability with combined cardiovascular end points, even after adjusting for covariates, including hypoglycemia episodes.

### Limitations

This study has limitations. While it adds an important association of longitudinal HbA_1c_ variation with major adverse cardiac and cerebrovascular events in patients with diabetes and multivessel CAD during a long-term follow-up period, the study of postbaseline factors, such as HbA_1c_ values, and their possible associations with the occurrence of events is challenging. Unlike other studies that assessed HbA_1c_ parameters, such as average real variability or coefficient of variation, which assume a constant variation of HbA_1c_ among the longitudinal measurements in time, the present study used a model that joins the information of a longitudinal outcome that is not constant in time with the occurrence of time-to-event data. In this way, the use of a joint model aiming to capture the association of varying factors with clinical end points strengthens the results of the present study. On the other hand, the retrospective nature of this analysis imposes limitations, especially because of the large number of patients who were excluded because of missing data. Moreover, information about other characteristics of diabetes could help to clarify the mechanisms of the underlying variation of HbA_1c_ levels. However, this study was not designed to assess these questions.

This study suggests that the control of glycemia and, consequently, HbA_1c_ should focus not only on achieving strict, isolated levels but also on minimizing variation over time. Especially in this population with multivessel CAD, in which control of diabetes might influence the occurrence of cardiac events, avoiding variation of HbA_1c_ levels could have the potential to lower cardiovascular risk during a long-term follow-up period.

## Conclusions

In this study, variation of HbA_1c_ levels was independently associated with the development of major cardiovascular events in patients with type 2 diabetes and multivessel CAD during a long-term follow-up period. The mechanisms underlying this association require further investigation.

## Notes

Supplement. **eTable 1. **Baseline Characteristics of Patients Included and Excluded From the Analysis **eTable 2. **Results of the Joint Models of the Association of HbA_1c_ Longitudinal Values With the Risk of Composite Cardiovascular Events **eTable 3. **Results of the Comparison of the Joint Models for the Association of HbA_1c_ Longitudinal Values With the Risk of Composite Cardiovascular Events
