# Supplementary material for: Association of Longitudinal Values of Glycated Hemoglobin With Cardiovascular Events in Patients With Type 2 Diabetes and Multivessel Coronary Artery Disease
Source: JAMA Netw Open. 2020 Jan 22;3(1):e1919666. doi: 10.1001/jamanetworkopen.2019.19666 (PMC6991270; doi:10.1001/jamanetworkopen.2019.19666)
Supplement: Supplement. — eTable 1. Baseline Characteristics of Patients Included and Excluded From the Analysis eTable 2. Results of the Joint Models of the Association of HbA1c Longitudinal Values With the Risk of Composite Cardiovascular Events eTable 3. Results of the Comparison of the Joint Models for the Association of HbA1c Longitudinal Values With the Risk of Composite Cardiovascular Events [file jamanetwopen-3-e1919666-s001.pdf]

# Supplementary Online Content

Rezende PC, Hlatky MA, Hueb W, et al. Association of longitudinal values of glycated hemoglobin with cardiovascular events in patients with type 2 diabetes and multivessel coronary artery disease. *JAMA Netw Open*. 2020;3(1):e1919666. doi:10.1001/jamanetworkopen.2019.19666

**eTable 1.** Baseline Characteristics of Patients Included and Excluded From the Analysis
**eTable 2.** Results of the Joint Models of the Association of $HbA_{1c}$ Longitudinal Values With the Risk of Composite Cardiovascular Events
**eTable 3.** Results of the Comparison of the Joint Models for the Association of $HbA_{1c}$ Longitudinal Values With the Risk of Composite Cardiovascular Events

This supplementary material has been provided by the authors to give readers additional information about their work.

© 2020 Rezende PC et al. *JAMA Network Open.*

eTable 1. Baseline Characteristics of Patients Included and Excluded From the Analysis

| | Included in the analysis (n=725) | Excluded of the analysis (n=163) | P |
|---|---|---|---|
| Age (years), median (range) | 62.4 (55.7-68.0) | 62.2 (54.7-68.7) | 0.72 |
| Male, n (%) | 467 (64.4) | 74 (64.3) | 0.98 |
| Hypertension, n (%) | 534 (75.5) | 88 (83.0) | 0.16 |
| Smoking, n (%) | | | 0.60 |
| Current | 119 (16.9) | 19 (17.8) | |
| No | 583 (83.1) | 88 (82.2) | |
| CKD (Creat > 1.5mg/dL), n (%) | 37 (5.7) | 10 (6.9) | 0.58 |
| EF (%), median (range) | 65 (60-70) | 65 (54-70) | 0.25 |
| CAD, n (%) | | | 0.42 |
| 2-vessel | 204 (31.6) | 39 (35.4) | |
| 3-vessel | 442 (68.4) | 71 (64.6) | |
| CAD treatment, n (%) | | | 0.06 |
| MT | 203 (28.1) | 43 (36.7) | |
| CABG | 328 (45.4) | 53 (45.3) | |
| PCI | 192 (26.5) | 21 (18.0) | |
| LDL-cholesterol (mg/dL), median (range) | 113 (89-144) | 137 (118-161) | <.001 |
| HbA1c (%, baseline), median (range) | 7.5 (6.4-9.2) | 8.1 (7.2-9.6) | 0.11 |
| Creatinine (mg/dL), median (range) | 1.0 (0.89-1.20) | 1.0 (0.90-1.20) | 0.07 |

CKD stands for chronic kidney disease; EF, ejection fraction; CAD, coronary artery disease; MT, medical therapy; CABG, coronary artery bypass grafting; PCI, percutaneous coronary intervention; LDL, low-density cholesterol.

© 2020 Rezende PC et al. *JAMA Network Open.*

eTable 2. Results of the Joint Models of the Association of $HbA_{1c}$ Longitudinal Values With the Risk of Composite Cardiovascular Events

| Parameters | HR (95% CI) | P |
|---|---|---|
| Unadjusted Model | 1.14 (1.04-1.24) | 0.002 |
| Adjusted Model* | 1.22 (1.12-1.35) | <.001 |
| Model B** | 1.25 (1.12-1.36) | <.001 |
| Model C*** | 1.24 (1.09-1.34) | 0.001 |

HR stands for hazard ratio; CI, confidence interval.

*Model adjusted for age, sex, ejection fraction, 2-3 vessel coronary artery disease, initial coronary artery disease therapy, creatinine, LDL-cholesterol.

**Multivariate model using time as a linear term.

***Multivariate model adding a quadratic term of time.

© 2020 Rezende PC et al. *JAMA Network Open.*

eTable 3. Results of the Comparison of the Joint Models for the Association of $HbA_{1c}$ Longitudinal Values With the Risk of Composite Cardiovascular Events

| Parameters | AIC | BIC | Log.Lik |
|---|---|---|---|
| Unadjusted Model | 19703.15 | 19798.44 | -9825.13 |
| Adjusted Model* | 19678.23 | 19801.15 | -9807.17 |
| Model B** | 20030.05 | 20132.73 | -9993.87 |
| Model C*** | 19820.54 | 19934.48 | -9875.22 |

The lower the levels of AIC and BIC, the higher the performance of the model.

*Model adjusted for age, sex, ejection fraction, 2-3 vessel coronary artery disease, initial coronary artery disease therapy, creatinine, LDL-cholesterol.

**Multivariate model using time as a linear term.

***Multivariate model adding a quadratic term of time.

© 2020 Rezende PC et al. *JAMA Network Open.*
